# MRI Revealing Ovarian Non-salvageable Torsion Masquerading as a Pelvic Mass in a Young Woman With Enterocystoplasty and Neurogenic Bladder

**DOI:** 10.7759/cureus.82104

**Published:** 2025-04-11

**Authors:** Hamza Retal, Soumya El Graini, Ibrahima Diallo Dokal, Ouadie El Menaoui, Jamal El Fenni

**Affiliations:** 1 Radiology Department, Ibn Sina University Hospital, Rabat, MAR; 2 Radiology Department, Mohammed V Military University Hospital, Rabat, MAR

**Keywords:** acute pelvic pain, ovarian torsion, pelvic mri, pelvic ultrasound, spina bifida

## Abstract

Adnexal torsion is a gynecological emergency requiring prompt diagnosis and intervention to prevent irreversible ovarian damage. This case report presents a 19-year-old female with a history of spina bifida, neurogenic bladder managed by enterocystoplasty, and chronic urinary retention, who presented with fluctuating pelvic and lumbar pain. Initial ultrasound showed bilateral hydronephrosis without obstruction, while subsequent imaging revealed a pelvic mass with features concerning for ovarian torsion. A non-contrast MRI confirmed a hemorrhagic, non-viable ovary with peripheral microfollicules and a twisted vascular pedicle, establishing the diagnosis of adnexal torsion with infarction. Laparoscopic surgery confirmed the torsion, necessitating oophorectomy due to irreversible ischemic damage. This case underscores the diagnostic challenges posed by atypical presentations, particularly in patients with pre-existing urological conditions. The differential diagnosis includes hemorrhagic ovarian cysts, endometriomas, ectopic pregnancy, and tubo-ovarian abscess, which can mimic torsion. Imaging plays a crucial role in differentiating these conditions and assessing ovarian viability. This report highlights the importance of a structured diagnostic approach, integrating ultrasound, CT, and MRI to optimize timely intervention and preserve ovarian function whenever possible. Radiologists should maintain a high index of suspicion in atypical cases to avoid delays in management and improve patient outcomes.

## Introduction

Ovarian torsion is a gynecological emergency that requires prompt recognition and surgical intervention to prevent irreversible damage to the ovary. It occurs when an ovary twists around its supporting ligaments, compromising its blood supply. Torsion typically affects women of reproductive age, and its presentation can be variable, often causing diagnostic challenges. Adnexal torsion accounts for 2-3% of acute pelvic pain cases and is most common in women under 20, with the right ovary being more frequently affected than the left due to its anatomical positioning. A significant risk factor is the presence of a benign ovarian mass, particularly cysts larger than 5 cm, which can increase the likelihood of torsion [1].

Clinically, ovarian torsion often presents with sudden, severe, and unilateral pelvic pain localized to the side of the affected ovary, frequently accompanied by nausea and vomiting. However, symptoms can be nonspecific and may overlap with other causes of acute abdominal pain, such as appendicitis or ectopic pregnancy, making clinical diagnosis challenging. For radiologists, the presence of an ovarian mass or cyst should raise suspicion for torsion, particularly if associated with characteristic imaging findings.

Ultrasound, the first-line imaging modality, plays a crucial role in detecting ovarian torsion. Key sonographic features include an enlarged, edematous ovary, peripherally displaced follicles, and the "whirlpool sign," which indicates a twisted vascular pedicle. Doppler ultrasound may show absent or reduced venous and arterial flow, but normal vascularity does not exclude torsion. In cases where ultrasound is inconclusive, further imaging with CT or MRI is recommended. MRI, in particular, provides high diagnostic accuracy, demonstrating findings such as ovarian stromal edema, hemorrhagic infarction, and the "whirlpool sign," aiding in distinguishing salvageable from non-salvageable ovaries [1,2].

Recognizing the imaging hallmarks of ovarian torsion in patients with an adnexal mass is critical for early diagnosis and timely intervention. Given the potential for ovarian loss and subsequent infertility, rapid imaging assessment and surgical management are essential for optimizing patient outcomes.

## Case presentation

A 19-year-old female with a history of spina bifida previously operated on, a neurogenic bladder managed by enterocystoplasty, and chronic urinary retention requiring an indwelling catheter presented to the emergency department with fluctuating pelvic pain localized to the right iliac fossa and left lumbar pain. The pain had persisted for 15 days, was intermittent and spontaneously resolving, with acute worsening 5 days prior, especially in the lumbar region. The patient denied systemic symptoms such as fever, nausea, vomiting, or recent urinary tract complaints.

On physical examination, the patient was hemodynamically stable, afebrile, and exhibited no signs of systemic infection. An abdominal examination revealed tenderness localized to the left iliac fossa without peritoneal signs such as guarding or rigidity. A neurological assessment showed no new lower limb deficits or sensory changes. Urinary evaluation confirmed the presence of an indwelling catheter without associated dysuria or hematuria.

An ultrasound performed five days prior to admission revealed bilateral hydronephrosis without visible obstruction and no appendiceal abnormalities. Despite the presence of hydronephrosis, no obvious urinary tract dilation or calculus was detected, and the bladder appeared unremarkable apart from chronic catheterization. Laboratory findings showed moderate leukocytosis with a white blood cell count of 12,000 G/L, while inflammatory markers remained within normal limits (C-reactive protein: 0.5 mg/L), suggesting the absence of significant systemic infection. Renal function tests, however, showed a slight worsening compared to the patient’s baseline, likely reflecting the progression of her chronic kidney disease. Given this deterioration, the decision was made to avoid contrast administration for both CT and MRI studies as a precautionary measure.

**Figure 1:**
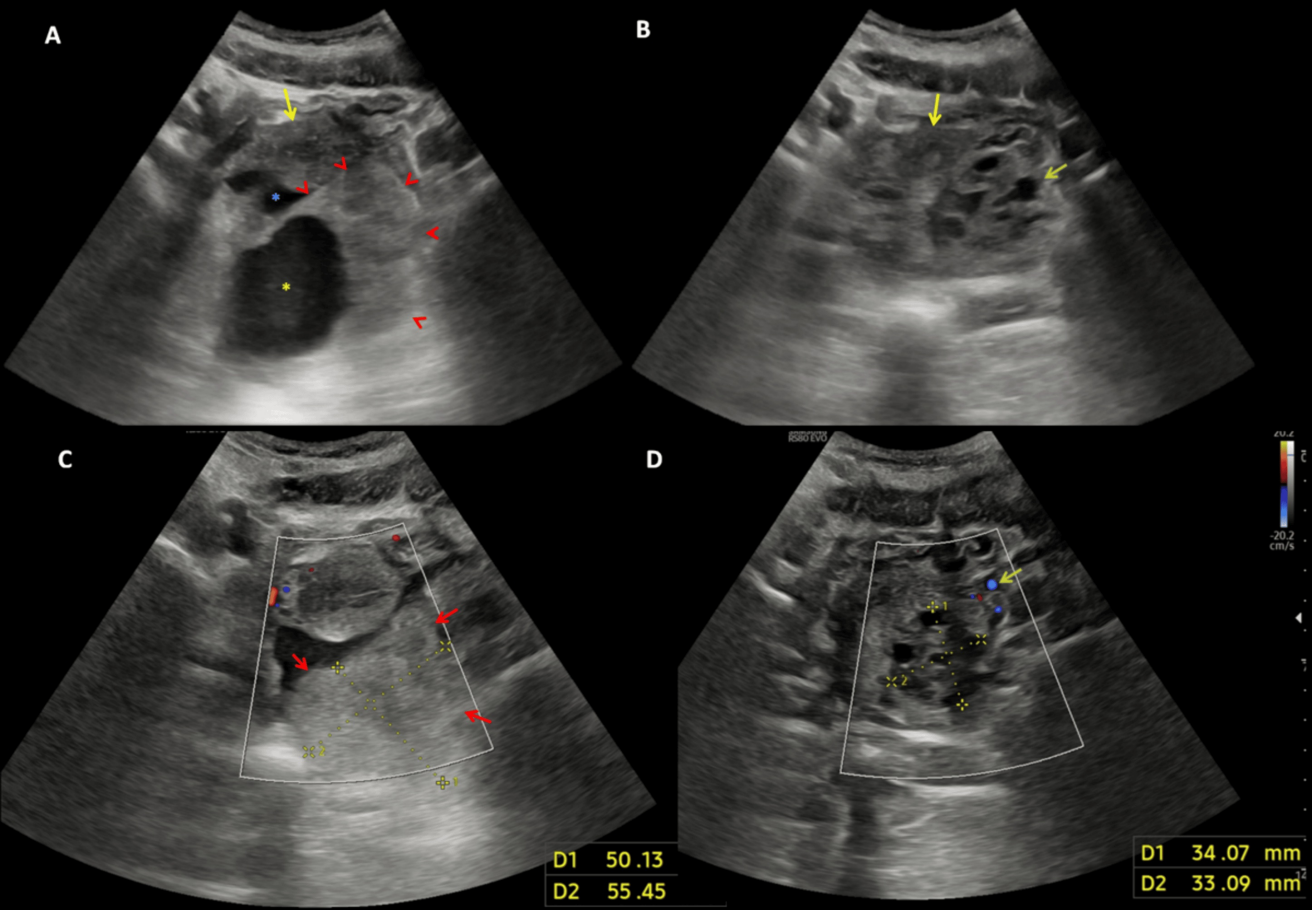
Ultrasound assessment of the pelvic mass A. Ultrasound image demonstrating a midline retro-uterine pelvic mass (red arrowheads), slightly deviated to the left, with a reniform shape and heterogeneous echotexture. The mass contains an internal cystic component (yellow asterisk) and is associated with a moderate volume of free pelvic fluid (blue asterisk). The uterus exhibits normal morphology (yellow arrow). B. Corresponding image delineating the uterus (yellow arrow) and a normal-sized left ovary with a follicular pattern (green arrow). C & D. Color Doppler imaging reveals an absence of vascular flow within the pelvic mass (red arrows), whereas the left ovarian vascular pedicle demonstrates preserved perfusion (green arrow). The right ovary and its vascular pedicle are not visualized.

Given the persistence of symptoms and the evolving nature of her pain, further imaging was warranted. A pelvic ultrasound performed in the emergency department revealed a heterogeneous echoic pelvic mass with a central cystic component. No clear vascular flow was detected on Doppler imaging. The left ovary was follicular and normal, raising concerns for right ovarian torsion or an adnexal mass compressing the left ureter. Bilateral pyelocaliceal dilation was found marked at the left side, in association with a normal enterocystoplasty and a urinary catheter within the bladder. Free pelvic fluid was also noted (Figure 1).

**Figure 2:**
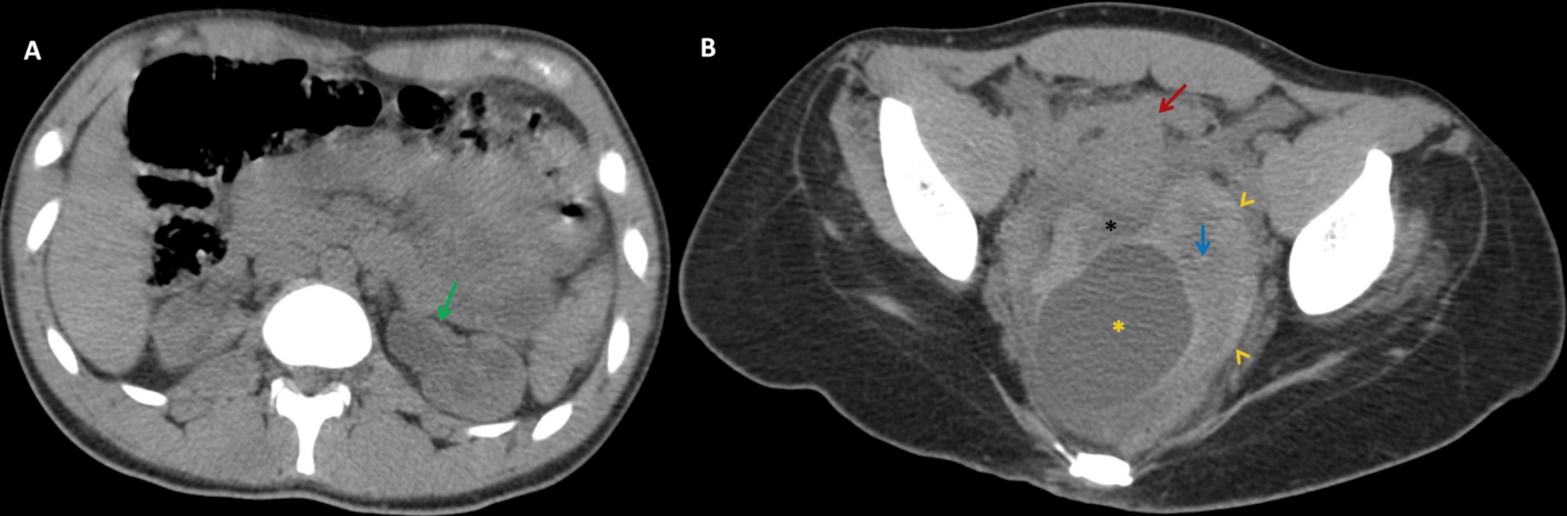
Axial non-contrast CT imaging of a pelvic mass and left urinary tract dilation A. Axial CT slice at the level of the renal hilum demonstrating left pyelocaliectasis (green arrow), indicative of upper urinary tract dilation. B. Axial CT slice at the pelvic level revealing the previously identified ultrasound mass, appearing as a reniform structure mimicking an ectopic kidney. The lesion exhibits a spontaneously hyperdense periphery (yellow arrowheads) and a hypodense central region (blue arrow), with an associated large cystic component (yellow asterisk). The uterus is visualized anterior to the mass (red arrow), along with free pelvic fluid (black asterisk).

To further characterize the lesion, a non-contrast computed tomography (CT) scan was performed. The CT confirmed the presence of a pelvic mass with a spontaneously hyperdense peripheral rim. Initially, it was thought to represent a pelvic kidney due to its location and shape. However, its internal cystic structure raised suspicion for adnexal pathology. Additionally, the right ovary was not visualized, while both kidneys were in their normal positions. Precise tissue differentiation remained challenging in this CT with no contrast injection (Figure 2).

**Figure 3:**
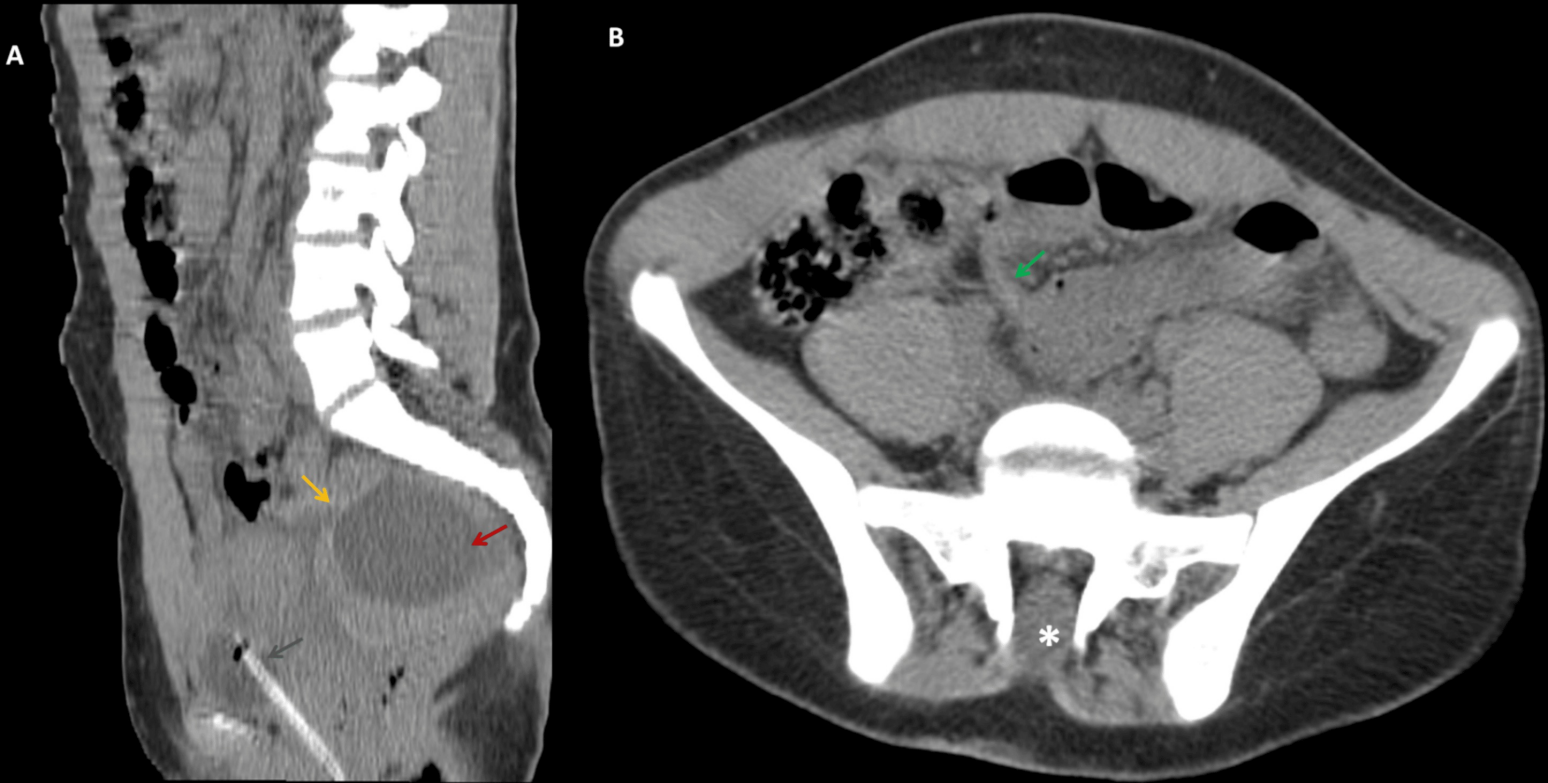
Non-contrast CT imaging of a pelvic mass with a normal appendix A. Sagittal view of a non-contrast CT scan, showing the previously described pelvic mass (orange arrow) with a central cystic lesion within it (red arrow). Note the urinary bladder catheter in position (blue arrow). B. Axial abdominal CT slice demonstrating a normally shaped appendix (green arrow). Additionally, note the posterior spinal defect (white asterisk).

The uterus was slightly deviated to the right, a finding often associated with adnexal torsion. Furthermore, pyelo-ureteral dilation was noted and is believed to result from the mass effect exerted by the pelvic lesion as the ureter traverses through it. No evidence of bowel pathology, appendiceal inflammation, or other intra-abdominal abnormalities was observed (Figure 3).

At this stage, imaging was highly suggestive of ovarian torsion with an intralesional cyst and hemorrhagic stroma, indicative of consumed ischemia and non-salvageable torsion. This was reinforced by a decrease in pain compared to the initial presentation.

**Figure 4:**
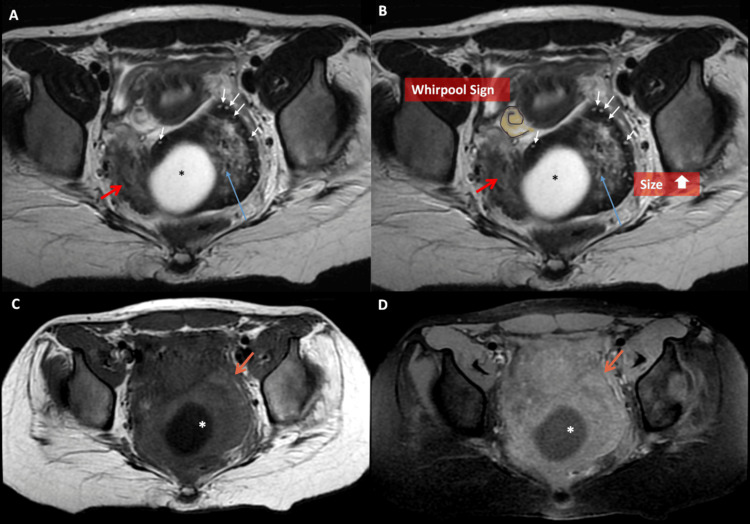
Axial T2 and T1-weighted MRI of a pelvic mass A & B. Axial T2-weighted images demonstrate a pelvic mass consistent with a tumefactive right ovary, exhibiting an increase in size. The mass is characterized by a prominent hypointense peripheral rim, containing numerous microfollicles, which appear hyperintense on T2 (white arrows). Additionally, there is stromal edema, which appears hyperintense (blue arrow), and a central simple cystic structure (black asterisk). Notably, the "whirlpool sign" is evident, as shown schematically in B (yellow form), along with a thickening of the fallopian tube wall (red arrow). C & D. Axial T1-weighted images, before (C) and after fat saturation (D), further confirm the cystic nature of the ovarian lesion (white asterisk). The slight peripheral rim hyperintensity (orange arrow) suggests a hemorrhagic transformation within the ovary.

To refine the diagnosis and assess ovarian viability, a non-contrast magnetic resonance imaging (MRI) scan of the pelvis was subsequently performed. The MRI confirmed ovarian torsion, revealing a hemorrhagic, non-viable right ovary with peripheral microfollicles that appeared hyperintense on T2-weighted images within a hypointense peripheral parenchyma. A central tumefactive stroma exhibited a hypointense signal on T1-weighted imaging and hyperintensity on T2, suggestive of edema, displacing follicles to the periphery within a mildly hemorrhagic peripheral ovarian rim, which appeared hyperintense on T1 and markedly hypointense on T2. The ovarian stroma appeared markedly edematous, with no diffusion restriction. The classic "whirlpool sign" of a twisted vascular pedicle, which was not visible on ultrasound or CT, further confirmed torsion on MRI, along with thickening of the fallopian tube wall. The enlarged right ovary was displaced medially, positioned just above the normal left ovary, forming the "kissing ovary sign" usually seen in deep pelvic endometriosis. The cystic mass within the right ovary appeared hypointense on T1 and brightly hyperintense on T2, measuring 62 × 47 mm, with no chocolate appearance or other signs of pelvic endometriosis (Figure 4).

**Figure 5:**
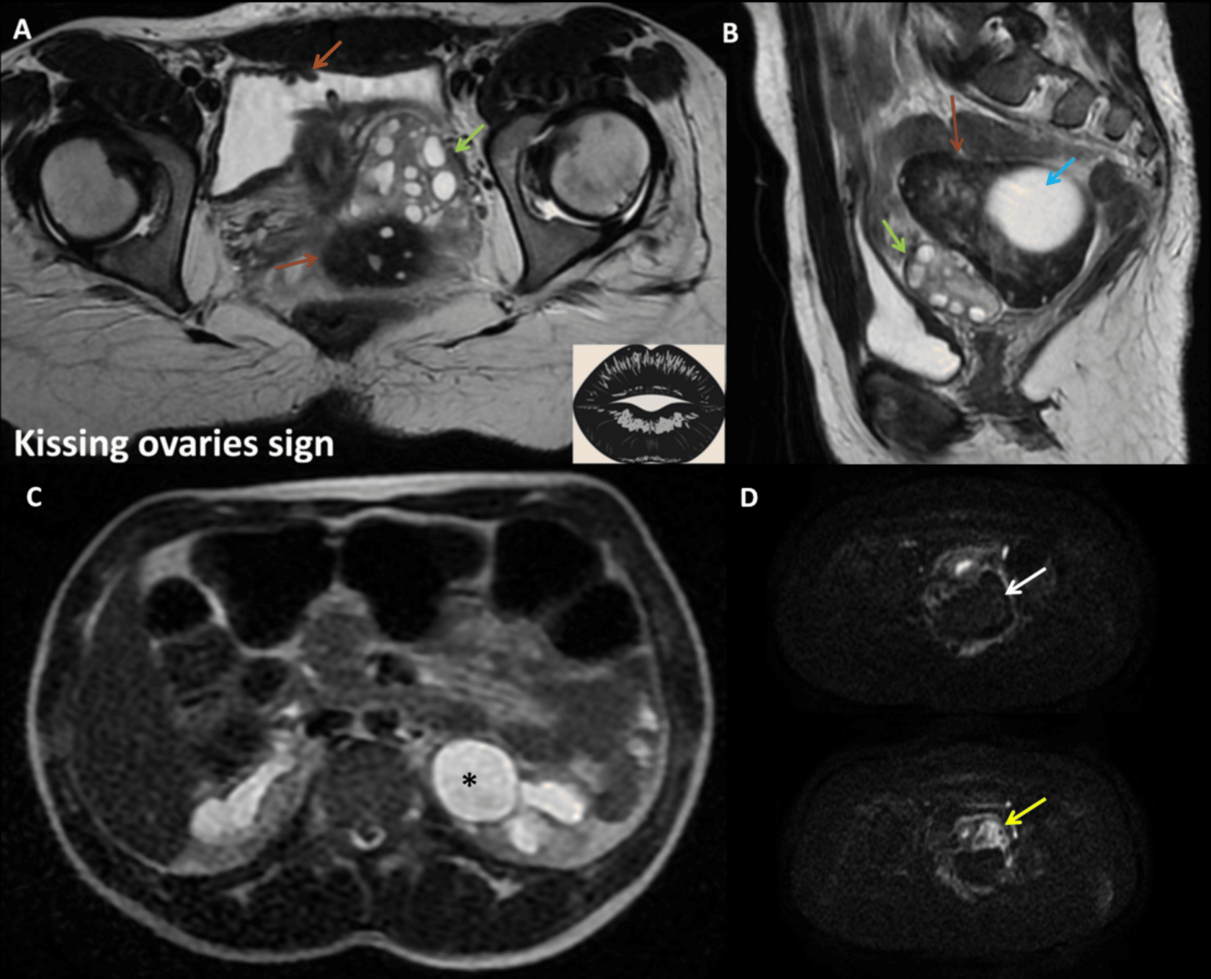
Pelvic MRI assessing the ovary and urinary tract dilation A. Axial T2-weighted MRI demonstrates a normal left ovary containing functional follicles, with a normal isointense stromal signal (green arrow), and a medially displaced right ovary that is markedly hypointense on T2-weighted imaging (red arrow), forming the "kissing ovary sign." Note the normal enterocystoplasty (orange arrow). B. Sagittal T2-weighted MRI shows the enlarged right ovary (red arrow), which retains its reniform morphology with a peripheral hypointense rim and a central heterogeneously hyperintense stroma, reinforcing the suspicion of an edematous stroma. The cystic lesion remains prominent within the right ovary (blue arrow). The left ovary appears normal in size, with multiple follicles and a normal stromal signal (green arrow). C. Axial abdominal T2-weighted imaging demonstrates left pelvic and caliceal dilation (black asterisk). D. Axial diffusion-weighted imaging (DWI) at a b-value of 1200 shows no T2 shine-through hyperintense effect or restricted diffusion within the right ovary (white arrow), while the left ovary exhibits normal diffusion-related hyperintensity (yellow arrow).

The initial misinterpretation of the mass as a pelvic kidney on ultrasound was clarified by MRI, which revealed a structure with ovarian morphology, cystic degeneration, and a twisted pedicle. Notably, the ultrasound examination took approximately 30 minutes, while the MRI was slightly faster, lasting 21 minutes from door to completion, and did not require a gadolinium injection. No signs of pelvic inflammatory disease or tubo-ovarian abscess were observed. These findings confirmed the diagnosis of ovarian torsion. Diffusion-weighted imaging (DWI) showed a normal left ovary while the affected ovary appeared markedly hypointense with high ADC values of 2.0 x10^-3^ mm^2^/s (Figure 5).

The final diagnosis was ovarian torsion with a mild hemorrhagic infarct due to a voluminous cyst, initially assessed as non-salvageable based on MRI findings, along with hydronephrosis likely caused by direct compression or autonomic dysfunction secondary to the neurogenic bladder. Despite the absence of diffusion restriction, which raised hope for a potentially viable ovary, surgical intervention was deemed necessary. The patient underwent an emergency laparoscopy, which confirmed a torsed, hemorrhagic right ovary. Due to the extent of ischemic damage, the ovary was ultimately deemed non-salvageable, necessitating an oophorectomy. The postoperative course was uneventful, with the resolution of lumbar pain. In conclusion, the lack of diffusion restriction may indicate prolonged ischemia, as symptoms had begun 15 days before the MRI assessment.

This case highlights the crucial role that imaging plays in assessing ovarian viability, prognosis, and salvageability. Early imaging and prompt surgical intervention are essential to prevent complications as seen in our patient and improve patient outcomes.

## Discussion

Adnexal torsion is a time-sensitive diagnosis that requires prompt recognition and intervention to prevent irreversible ovarian damage. However, it remains a diagnostic challenge due to its variable clinical presentation and overlapping imaging features with other gynecologic and non-gynecologic conditions. The reported case highlights the complexity of diagnosing adnexal torsion, particularly in young patients with non-specific symptoms. While lower abdominal pain is a hallmark feature, its intermittent nature and the absence of systemic signs often delay suspicion. This aligns with findings emphasizing that adnexal torsion is primarily a vascular compromise rather than an inflammatory process, making standard inflammatory markers less useful.

Adnexal torsion accounts for 2%-3% of acute pelvic pain cases in females, and about 30% of them occur in patients less than 20 years old. The single greatest risk factor is the presence of a benign ovarian lesion, found in over 80% of cases. Ovarian masses >5 cm significantly increase the risk of torsion. The right ovary is more frequently affected than the left due to the anatomical position of the sigmoid colon stabilizing the left ovary [1,2].

Imaging plays a crucial role in confirming the diagnosis, as clinical examination alone is often insufficient. The challenge lies in distinguishing adnexal torsion from other conditions with similar presentations such as hemorrhagic ovarian cysts, endometriomas, tubo-ovarian abscesses, and ectopic pregnancy. A structured imaging approach is essential for accurate differentiation [3].

Ultrasound (US), particularly transabdominal and transvaginal Doppler US, if feasible, is the first-line modality for suspected adnexal torsion. Key findings include an enlarged ovary with a peripheral displacement of follicles, a twisted vascular pedicle (“whirlpool sign”), and Doppler flow abnormalities. An enlarged ovary is seen in 53%-85% of cases, the twisted vascular pedicle in 79%, and peripheral small follicles in 12%-38%. However, ultrasound has limitations, as incomplete torsion may preserve arterial flow initially, leading to false-negative results. Additionally, those findings, as in our case, aren’t always seen. If the ultrasound is inconclusive, further imaging with CT or MRI is warranted [1,3].

CT, though not the primary modality, is often performed in emergency settings when the clinical presentation is unclear. Findings suggestive of adnexal torsion include unilateral ovarian enlargement with peripheral follicles, the whirlpool sign, uterine deviation toward the twisted adnexa, and ascites in cases with infarction. An enlarged midline ovary is seen in 87%-100% of cases, and the uterus is pulled toward the affected side in 36%-68%. A major diagnostic pitfall is mistaking an infarcted ovary for a pelvic mass, as an ischemic ovary can appear solid and heterogeneous, mimicking ovarian malignancy. Recognizing the twisted pedicle and correlating with clinical findings is crucial to avoid unnecessary oncologic workup [1,3].

When adnexal torsion is suspected, but ultrasound and CT findings are inconclusive, MRI provides the highest diagnostic confidence. MRI findings include ovarian stromal edema (hyperintense on T2-weighted images), hemorrhagic infarction (hyperintense on T1-weighted images with no enhancement), the whirlpool sign (best seen on 3D sequences), and lack of contrast enhancement, confirming nonviable ovarian tissue. MRI findings show peripheral hyperintense follicles on T2-weighted imaging in 27%-86% of cases and twisted pedicle in 14%-70%. Absent contrast enhancement suggests non-viability (seen in 17% of cases). MRI is particularly useful in distinguishing salvageable ovaries from infarcted ones, guiding management decisions. If any degree of enhancement is preserved, conservative detorsion may be considered, whereas a complete lack of enhancement suggests irreversible infarction, necessitating oophorectomy [1,4].

Additional MRI findings that further aid in diagnosing ovarian torsion include ovarian displacement, often seen in the pouch of Douglas, anterior to the uterus, or at midline. This displacement may hinder visualization on ultrasound and CT, making MRI particularly useful in such cases. Another significant MRI feature is the follicular ring sign, where peripheral displacement of follicles due to edema creates a hyperintense ring on T2-weighted images, a reliable predictor of torsion. However, this can also be observed in polycystic ovaries [4].

The whirlpool sign, resulting from the twisted vascular pedicle, remains a strong indicator of ovarian torsion and is best identified on MRI T2-weighted images. However, studies suggest it is seen in less than a third of cases on MRI. Additionally, MRI can identify modification of the ovarian margin, indicating pressure-related distortion of the ovarian wall [1].

Diffusion-weighted imaging (DWI) has proven to be increasingly valuable in assessing ischemic tissue, cytotoxic edema, and hemorrhages. Research suggests that DWI has a high sensitivity (94%) and specificity (100%) for differentiating ischemic from normal ovarian tissue. Hemorrhagic infarcts typically present as hypointense stroma with hyperintense fluid in the pelvic recess on DWI, often accompanied by low ADC values. This imaging modality helps predict ovarian salvageability before surgical intervention, particularly in cases where conventional imaging may be inconclusive [5,6].

In our case, however, despite the absence of diffusion restriction, which typically suggests ischemic damage, we initially held hope that the ovary might still be viable. This finding is intriguing because, in the early stages of torsion, we would typically expect to see restricted diffusion, indicating ischemia, as found in some literature studies and case reports [7,8]. However, the lack of diffusion restriction in this case, coupled with the prolonged nature of the symptoms (15 days before the MRI), suggests that in later stages of ovarian torsion, diffusion restriction may be absent, potentially due to the ischemic damage having reached a more advanced stage.

Susceptibility-weighted imaging (SWI) is an emerging MRI technique, primarily used in neuroimaging, but is now being explored for its ability to detect hemorrhagic infarcts in ovarian torsion. SWI maps distortions in magnetic fields caused by hemosiderin or variations in hemoglobin composition. Although research is limited, SWI has demonstrated greater sensitivity than both CT and standard T2-weighted MRI in detecting hemorrhage and could become a future tool in diagnosing ovarian torsion [1].

Gadolinium-enhanced MRI has traditionally been considered useful in adnexal imaging, as many authors used the enhancement finding as a sign of viability of the ovary, but its role in evaluating ovarian torsion remains debated. Studies suggest that post-contrast sequences do not provide additional benefits in detecting acutely torsed ovaries, as torsion is primarily a vascular event identified through indirect signs such as stromal edema, lack of perfusion, and the whirlpool sign. However, contrast-enhanced MRI can improve specificity in distinguishing torsion from other adnexal pathologies, particularly when adnexal lesions are present. A study evaluating the utility of contrast-enhanced MRI in pediatric patients found that contrast administration helped correctly rule out torsion in cases with ovarian lesions in 29%-38% of cases, reducing false positives. However, it did not significantly change the detection rate of acute torsion itself. Given these findings, contrast-enhanced imaging should be individualized and reserved for cases with ambiguous ultrasound or pre-contrast MRI findings, particularly when distinguishing between torsion and other adnexal conditions [9]. In addition, a study suggests using MRI as a first-line imaging modality for evaluating ovarian torsion, offering a faster way to diagnose compared to ultrasound, which has a median scanning time of 24.1 minutes versus 11.4 minutes for MRI [6,10].

Adnexal torsion shares imaging features with several other conditions, making differentiation critical. Hemorrhagic ovarian cysts typically appear as hyper-echoic cysts with reticular internal echoes on ultrasound and as T1 hyperintense lesions with “shading” on T2 MRI, often presenting with acute pain if rupture occurs. Endometriomas, in contrast, exhibit homogeneous low-level echoes on ultrasound and T1 hyperintensity with T2 shading on MRI, usually seen in the context of chronic pelvic pain and dysmenorrhea. Ectopic pregnancy, another important differential, presents as an adnexal mass with trophoblastic tissue and associated hemoperitoneum, often accompanied by a positive beta-HCG test. Tubo-ovarian abscesses, typically seen in the setting of pelvic inflammatory disease, demonstrate multiloculated cystic structures with thick septations, rim enhancement, and surrounding inflammatory changes, often associated with fever and leukocytosis. Pelvic inflammatory disease (PID) itself manifests as thickened, hyperemic fallopian tubes with adjacent inflammatory findings, differentiating it from adnexal torsion, which primarily involves vascular compromise rather than inflammation [1,4].

To optimize diagnosis, a structured imaging algorithm is recommended. Ultrasound should be the first-line assessment, focusing on ovarian enlargement, peripheral follicles, and the whirlpool sign while recognizing the limitations of Doppler flow. If the ultrasound is inconclusive, an MRI scan should be performed to assess for ovarian enlargement, uterine deviation, and hemorrhagic infarction while ruling out mimicking conditions (Table 1)

**Table 1:** Radiological findings in ovarian torsion

Category	Findings
Clinical Findings	Sudden onset of severe, unilateral pelvic pain (most commonly on the right side): Nausea and vomiting - Tenderness on pelvic examination - Possible low-grade fever (in cases of infarction) - History of intermittent pain (intermittent torsion)
Ultrasound Findings	Enlarged ovary (> 4 cm) with peripheral displacement of follicles - "Whirlpool sign" (twisted vascular pedicle) - Free fluid in the pelvis - Doppler abnormalities: Absent or reduced venous and arterial flow (but normal flow does not exclude torsion)
CT Findings	Enlarged ovary with perifollicular edema - Deviation of uterus toward the twisted ovary - Thickened fallopian tube - Free pelvic fluid
MRI Findings	T2-weighted imaging: Stromal edema (hyperintense signal) - T1-weighted imaging: Hemorrhagic infarction (variable signal intensity) - Whirlpool sign confirming twisted pedicle - Deviation of ovarian ligaments and displacement of the ovary
Gadolinium-Enhanced MRI	Absent or diminished contrast enhancement in necrotic areas - Patchy or heterogeneous enhancement in viable ovarian tissue - Contrast enhancement of twisted pedicle (target sign)
Susceptibility-Weighted Imaging (SWI)	Hypointense signal indicating hemorrhage (suggesting infarction)
Diffusion-Weighted Imaging (DWI)	Restricted diffusion in ischemic areas (high DWI signal, low ADC values) - Can help differentiate between salvageable and non-salvageable ovaries

When torsion remains suspected, MRI provides the highest specificity, confirming viability through contrast-enhanced sequences. If enhancement is present, detorsion may be considered, whereas absent enhancement suggests the need for oophorectomy [6].

Physicians must maintain heightened awareness and an index of suspicion when approaching a woman with pain in any region of the abdomen or pelvis. Further investigation with abdominal and pelvic ultrasonography and magnetic resonance imaging or computed tomography is necessary. Benign lesions can be found even in patients presenting with giant masses and elevated CA-125 levels. Tumor markers, such as CA 125 and CA 19-9, play an essential role in the assessment of gynecological tumors, particularly in differentiating benign from malignant masses. However, their elevation is not exclusive to malignancy; it can also be seen in benign conditions such as endometriosis, pelvic inflammatory disease, and large ovarian cysts. Recent studies highlight the need for a multimodal approach combining imaging with tumor markers to improve diagnostic accuracy and avoid unnecessary surgical interventions [7-9]. Case reports have documented instances where elevated CA 125 and CA 19-9 levels were associated with benign conditions, such as uterine leiomyoma, serous cystadenoma, and ovarian mucinous borderline tumors, emphasizing the necessity of a thorough differential diagnosis before proceeding with invasive management [11-13].

Diagnosing adnexal torsion requires a high index of suspicion and a structured imaging approach to differentiate it from mimicking conditions. While ultrasound remains the first-line modality, it has limitations in detecting early torsion. CT provides valuable secondary signs, while MRI is the gold standard for assessing ovarian torsion. The combination of imaging modalities ensures accurate diagnosis, preventing unnecessary delays and optimizing surgical decision-making. Surgical detorsion is recommended for all cases before considering oophorectomy, even if the ovary appears non-viable. Early detection improves ovarian salvage rates, but delays lead to infarction. In children, surgical overestimation of non-viability is common, emphasizing the need for conservative management when feasible, as visual assessment of ovarian necrosis was not a good predictor of real necrosis in histopathology in some studies [14,15].

## Conclusions

Adnexal torsion remains a diagnostic challenge due to its variable clinical presentation and overlap with other pelvic pathologies. This case illustrates the importance of a structured imaging approach, particularly in patients with pre-existing urological conditions that may obscure classical symptoms. While ultrasound remains the primary tool for initial evaluation, MRI provides superior tissue characterization, enabling accurate differentiation between ovarian torsion and its mimics, as well as some viability signs. Diffusion-weighted imaging (DWI), in particular, plays a critical role in assessing ovarian viability and ischemic damage. However, this case highlights that the absence of diffusion restriction in the later stages of ovarian torsion may not necessarily indicate viability, as seen in our patient. The prolonged ischemic period before imaging could potentially explain this finding, suggesting that DWI results may be influenced by the timing of ischemic damage. Thus, further studies are needed to explore the role of DWI at different stages of ovarian torsion and its implications for ovarian salvageability.

Prompt diagnosis and surgical intervention are crucial in optimizing patient outcomes, as delayed recognition can lead to ovarian necrosis and loss of reproductive function. This case underscores the need for heightened awareness among radiologists and clinicians when evaluating patients with atypical presentations, ensuring timely diagnosis and appropriate management to preserve ovarian function whenever feasible.
